# Complexity of Factors Involved in Human Population Growth

**DOI:** 10.1289/ehp.112-1247545

**Published:** 2004-09

**Authors:** Larry Hobbs, Charles Fowler

**Affiliations:** Inland Whale, Bainbridge Island, Washington, E-mail: larry@inlandwhale.com; Systemic Management Studies Program, National Marine Mammal Laboratory, Seattle, Washington, E-mail: charles.fowler@noaa.gov

We would like to thank Bob Weinhold (2004) for his informative article documenting the issues facing humans in regard to infectious disease and the growing concern within the medical community that traditional thinking, approaches, and methods may well be inadequate to face the challenges ahead. We would also like to thank Steven Salmony (2004) for his thoughtful letter regarding Weinhold’s (2004) article, in which he presents another extremely important issue: that of human population growth and its interconnection with food resources. Both of these articles report the results of good science, and both describe well some of the critical issues facing humans at this time. We would like to present another viewpoint that we believe is both more helpful and more accurate for describing the problems we face and for setting research and decision-making goals that will involve the health of all systems.

Our approach (Fowler and Hobbs 2002, 2003) stems from systemic thinking as a paradigm that is emergent from modern systems theory, cybernetics, and information theory from their beginnings in the late 1940s. Basically, this way of thinking posits that all things are intricately interconnected in very complex ways, so that any action (or inaction, for that matter) will always result in a variety of consequences. Some of these we can predict and some we cannot; some will be evaluated as positive and some will be evaluated as negative in human value systems. Examples of these systemic reactions can be given for any field of inquiry (e.g., environmental, social, political, religious, personal) and at any level of organization (e.g., individual, species, ecosystem, biosphere); what we find is that there is never a single cause or a single outcome. It is always more complex than that. As humans, we have been able to ignore this complexity until very recently because simple cause-and-effect models were accurate enough to help us deal with the problems we faced.

However, as we have become more sophisticated with our technologies, we are experiencing unprecedented success at altering our world. The resulting changes are so profound that simple models no longer adequately describe the problems or define goals and guidelines to solve these problems. Certainly, as Hopfenberg (2003) so clearly pointed out, humans are biological organisms, and food availability is one of the factors that contribute to the wealth of factors that determine population size. It is, however, also true that the number of other factors that influence human population size is beyond human capacity to list, comprehend, and synthesize. We cannot measure them all nor can we accurately weigh the relative importance of each factor’s influence on the actual number of humans (e.g., disease, parasites, social upheaval, religious viewpoints, economics).

Each such factor is, in turn, influenced by other factors. For example, weather patterns influence the amount of food available. Ocean currents influence weather patterns; the orbiting of the earth and moon influence ocean currents; the orbits of other planets and the gravitational forces of the sun and other celestial bodies influence the orbits of the earth and moon; and so on. In each case, there are multitudes of other factors involved. The amount of food available is dependent on, or influenced by, microbes, other consumers, and predators and prey at all levels. A huge variety of physical forces is also at play in influencing primary and other levels of production, including volcanoes, hurricanes, floods, forest fires, and various human influences such as the use of pesticides and fertilizers and increased carbon dioxide production.

Human population numbers are also dependent on an enormous number of factors beyond food, including disease and all the other factors that were listed by Weinhold (2004). Had we been unable to curtail the effects of smallpox, for example, the human population would probably be smaller than it is today, as is the case for so many wildlife species whose populations are regulated, in part, by the effects of disease. However, when considering human population numbers, human value systems, economics, politics, and religion, all factors over which we have some limited measure of control, must also be taken into account.

We believe that any approach to dealing with human problems must take into account all of this complexity or it will lead to more problems. A systemic approach, such as we propose in our work (Fowler and Hobbs 2002, 2003), takes into account all of this complexity and also gives empirical guidelines for how to deal with the problems. It not only allows us to deal with how much food can be sustainably extracted from the various resource systems to feed ourselves but addresses the deeper and, we believe, more important question: how many of us should there be to feed?
